# Angiomotin stabilization by tankyrase inhibitors antagonizes constitutive TEAD-dependent transcription and proliferation of human tumor cells with Hippo pathway core component mutations

**DOI:** 10.18632/oncotarget.9117

**Published:** 2016-04-29

**Authors:** Albino Troilo, Erica K. Benson, Davide Esposito, Rachel-Ann A. Garibsingh, E. Premkumar Reddy, Sathish Kumar Mungamuri, Stuart A. Aaronson

**Affiliations:** ^1^ Department of Oncological Sciences, Icahn School of Medicine at Mount Sinai, New York, NY, USA

**Keywords:** tankyrase inhibitors, TEAD, YAP, angiomotin, tumor cell proliferation

## Abstract

The evolutionarily conserved Hippo inhibitory pathway plays critical roles in tissue homeostasis and organ size control, while mutations affecting certain core components contribute to tumorigenesis. Here we demonstrate that proliferation of Hippo pathway mutant human tumor cells exhibiting high constitutive TEAD transcriptional activity was markedly inhibited by dominant negative TEAD4, which did not inhibit the growth of Hippo wild-type cells with low levels of regulatable TEAD-mediated transcription. The tankyrase inhibitor, XAV939, identified in a screen for inhibitors of TEAD transcriptional activity, phenocopied these effects independently of its other known functions by stabilizing angiomotin and sequestering YAP in the cytosol. We also identified one intrinsically XAV939 resistant Hippo mutant tumor line exhibiting lower and less durable angiomotin stabilization. Thus, angiomotin stabilization provides a new mechanism for targeting tumors with mutations in Hippo pathway core components as well as a biomarker for sensitivity to such therapy.

## INTRODUCTION

The Hippo pathway is an evolutionarily conserved signaling pathway that plays a fundamental role in growth control, stem cell function, tissue regeneration, and tumor suppression [1, 2]. It features a core kinase module characterized by MST1/2 and LATS1/2 that phosphorylate and inhibit the transcriptional co-activators, YAP/TAZ, by preventing their nuclear localization [1]. YAP/TAZ lack an intrinsic DNA-binding domain and thus they can contact the DNA only through transcription factor partners such as TEAD1/−2/−3/−4, Runx1/−2, p73, Pax3, AP-1, or TBX5 [3]. Among these, TEAD family members appear to play a dominant role as primary mediators of YAP/TAZ-dependent gene regulation with target genes including a number involved in cell proliferation and cell motility [4–6].

YAP overexpression in model systems *in vivo* was initially shown to confer transforming, invasive, and prosurvival properties [7], which could be abrogated by YAP downregulation [8], and Hippo pathway alterations have increasingly been implicated in human tumorigenesis. In addition to YAP amplification or over expression observed in various epithelial malignancies [9] as well as YAP or TAZ translocations [9] or point mutation [10], loss of function mutations of core components of the Hippo inhibitory pathway such as LATS, or NF2 are found at high frequencies in mesotheliomas [11, 12]. Moreover, NF2 is commonly mutated in familial meningiomas and schwannomas as well as in spontaneous tumors of these and other tumor types [13]. Recent studies have identified GPCRs, which signal to either activate or inhibit Hippo signaling [14], and mutations in some G proteins have now been shown to activate YAP-dependent TEAD transcriptional activity in a high fraction of uveal melanomas and at lower frequency in other melanomas [15, 16]. Deep sequencing studies have revealed that almost 20% of human tumors harbor mutations in GPCRs [17], suggesting that mutations in other GPCRs and G proteins may also deregulate the Hippo pathway. Epigenetic silencing of Hippo components has been reported in human cancer as well [18–20].

The emerging role of Hippo pathway deregulation in cancer has increasingly focused attention on this signaling pathway as an anticancer target [1]. However, efforts focused on chemical inhibition of deregulated hippo signaling tumors are still in their infancy. In the present study, we genetically validated constitutive high TEAD-mediated transcription levels in human tumor cells with loss of function mutations in well-established Hippo pathway core components, LATS and NF2, as therapeutic targets and identified a mechanism by which small molecule tankyrase inhibitors specifically antagonize such Hippo pathway deregulated tumor cells.

## RESULTS

### Hippo pathway mutant tumor cells are reliant on high constitutive TEAD transcriptional activity for proliferation

**Figure 1:**
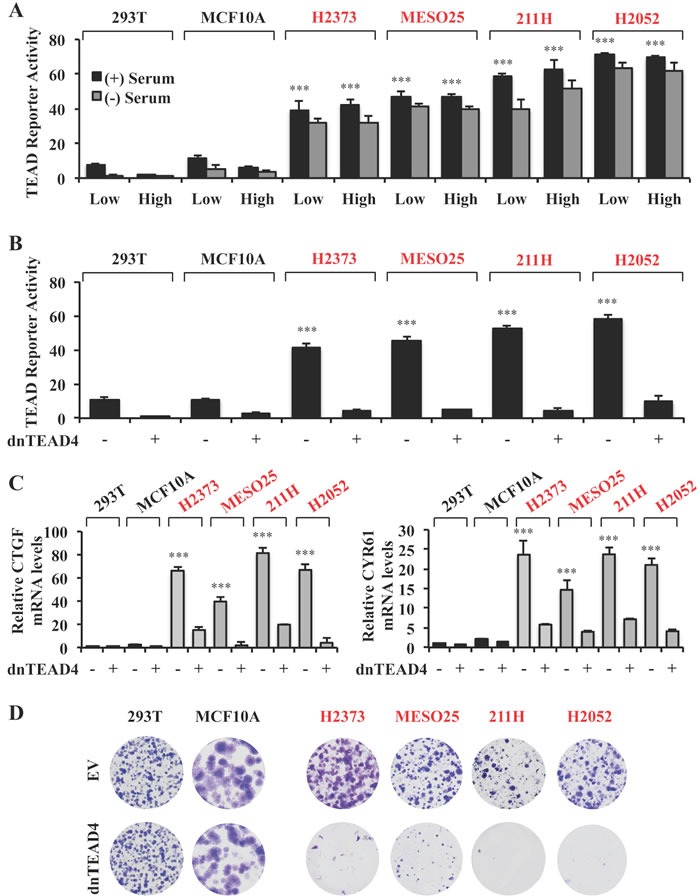
Hippo pathway mutant tumors are reliant on TEAD transcriptional activity for proliferation **A.** TEAD reporter activity in Hippo pathway wild-type (black) and mutant (red) cells. Cells were seeded at either low (2×10^4^ cells) or high (1.5x10^5^ cells) density in 24 well plates, in the absence or presence of 10% serum and the TEAD luciferase reporter was measured and normalized to the renilla luciferase in each cell line after 15 hours incubation. These values are shown as relative to those in 293T line cultured at low density and in the presence of serum. **B.**, **C.** TEAD reporter activities **B.** and mRNA expression levels relative to those in 293T empty vector **C.** in Hippo pathway wild-type and mutant cells stably expressing dnTEAD4. **D.** Representative images of colony formation by the cell lines as indicated in B. Error bars indicate standard deviation (SD) of experiments performed in triplicate. ***P≤0.001. Student *t-*Test.

The Hippo pathway regulates cell proliferation in response to cell density and external stimuli such as serum deprivation [14, 21, 22]. To characterize the effects of recurrent mutations in Hippo pathway core components in human tumor cells, we measured TEAD transcriptional activity in several tumor lines bearing loss of function mutations in NF2 (H2373, MESO25) [11], LATS1 (MSTO-211H (211H)) [23] and NF2/LATS2 (H2052) [11] or in immortalized non-tumorigenic (293T, MCF10A) cell lines, which are wild-type for NF2, LATS1 and LATS2 genes (Supplementary Figure S1A). Using a TEAD luciferase reporter assay, we observed that tumor lines harboring Hippo pathway mutations showed much higher reporter levels, which were insensitive to serum deprivation or high cell density as compared to Hippo pathway wild-type lines (Figure 1A). An antibody that recognizes both YAP and TAZ proteins detected higher YAP levels in each line. Of note, YAP protein levels were markedly higher in Hippo mutant as compared to wild-type cells despite their similar mRNA levels (Supplementary Figure S1A, S1B).

To determine how inhibition of TEAD-mediated transcription influenced cell proliferation, we stably expressed a dominant negative mutant form of TEAD4 (dnTEAD4) that is unable to interact with YAP to drive gene transcription [24] (Supplementary Figure S1C, S1E and S1G–S1J). Expression of dnTEAD4 effectively decreased TEAD reporter activity in both Hippo wild-type and mutant cells (Figure 1B). Moreover, expression levels of well-recognized TEAD target genes (CYR61 and CTGF) [14, 24] were significantly decreased under these conditions (Figure 1C; Supplementary Figure S1D, F). Of note, dnTEAD4 expression markedly inhibited the proliferation of Hippo mutant cell lines but had no detectable effect on colony formation by Hippo pathway wild-type lines (Figure 1D). These data demonstrate that tumor cells with loss of function mutations in the Hippo pathway core components were dependent on high TEAD transcriptional activity for their proliferation even in serum containing medium. In contrast, cells that lacked mutations in the pathway exhibited low, regulatable TEAD transcriptional activity, which was dispensable for their proliferation. Thus, we hypothesized that pharmacological inhibitors of TEAD transcriptional activity might specifically antagonize the transformed phenotype of Hippo pathway deregulated tumor cells.

### A small molecule screen identifies XAV939 as a novel inhibitor of TEAD transcriptional activity

**Figure 2:**
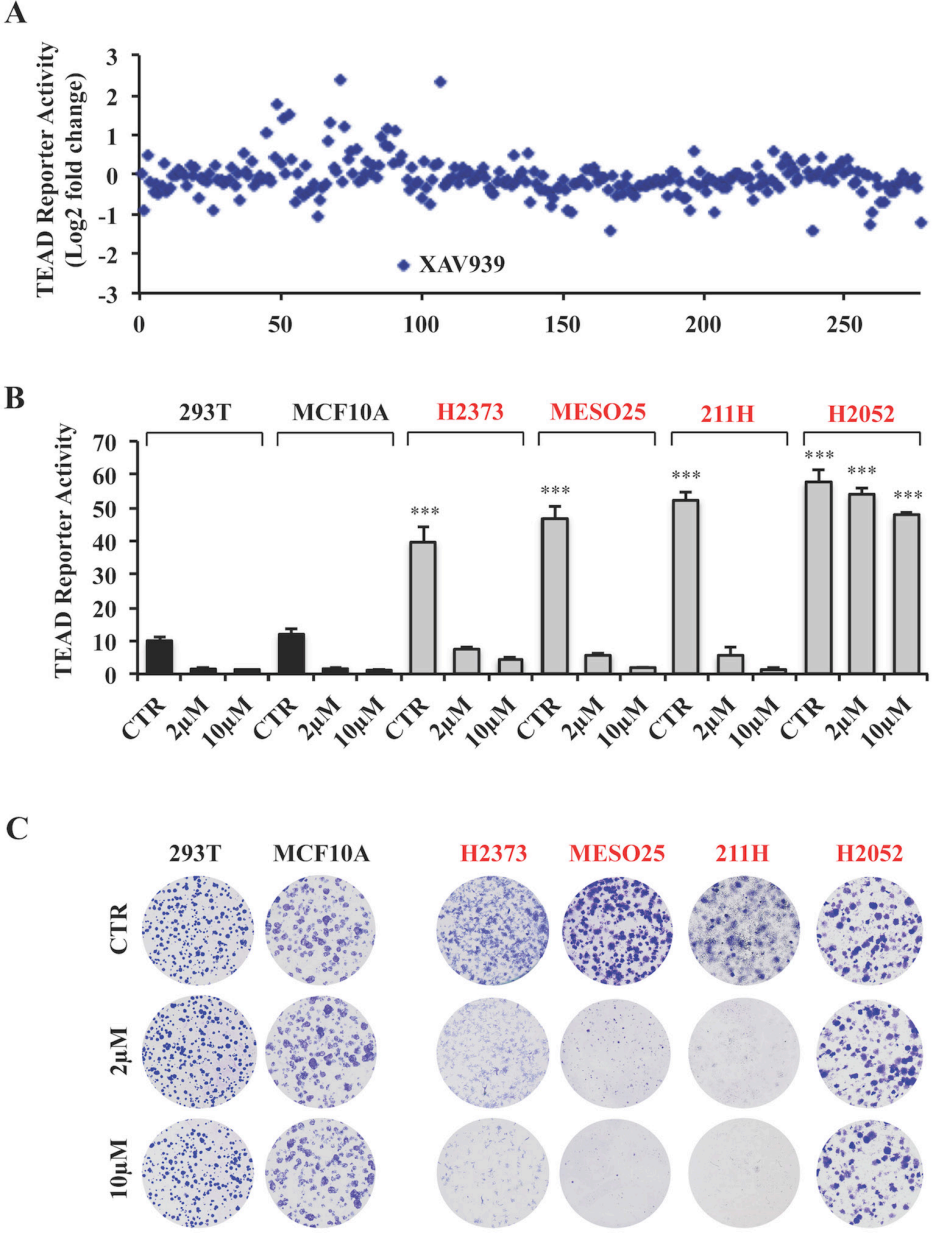
A small molecule screen identifies XAV939 as a novel inhibitor of TEAD transcriptional activity **A.** TEAD reporter activity of 293 cells treated for 24 hours with inhibitors at a concentration of 10μM. **B.** TEAD reporter activity of Hippo pathway wild-type and mutant cells treated with XAV939 or 0.1% DMSO as control (CTR) for 24 hours. **C.** Representative images of colony formation by the indicated cell lines treated with XAV939 or CTR. Error bars indicate SD of experiments performed in triplicate. ***P≤0.001. Student *t-*Test.

To search for small molecule inhibitors of TEAD transcriptional activity, we screened a library of in-house kinase and commercially available inhibitors by measuring their effect on TEAD reporter activity in 293T cells (Figure 2A). Whereas a few increased and 5 decreased the reporter activity by at least 50%, only one, XAV939, a tankyrase inhibitor initially identified as an inhibitor of Wnt signaling [25], decreased TEAD reporter activity by 75% (Figure 2A). Thus, we focused on investigating the effects of XAV939 on Hippo pathway mutant and non-mutant cells.

Similar to results with dnTEAD4 overexpression, XAV939 treatment markedly decreased TEAD reporter activity and the expression of TEAD target genes in all cell lines tested with the exception of H2052 cells (Figure 2B and Supplementary Figure S2A–S2F), in which the reporter and TEAD target gene expression were only modestly affected (Figure 2B and Supplementary Figure S2F). XAV939 treatment, as with dnTEAD4 overexpression (Figure 1D), had no effect on the proliferation of 293T and MCF10A (Figure 2C), nor was there any effect on the proliferation of 501T human diploid fibroblasts (data not shown). Whereas XAV939 markedly inhibited the proliferation of Hippo pathway mutant H2373, MESO25 and 211H, it had no effect on H2052 cells (Figure 2C), whose colony forming ability like that of the other Hippo mutant tumor lines was strongly inhibited by dnTEAD4 (Figure 1D). Cell cycle analysis further revealed that those tumor lines whose proliferation was inhibited, showed increased G1 and reduced S phase fractions without an obvious increase in apoptosis while there was no detectable cell cycle alteration in those, which were not growth inhibited (Supplementary Figure S3). These results demonstrated that XAV939 phenocopied the G1 arrest induced by dnTEAD4 in Hippo mutant tumor lines that were sensitive to XAV939-mediated inhibition of TEAD transcriptional activity.

### XAV939 regulates TEAD transcriptional activity through tankyrase inhibition

**Figure 3:**
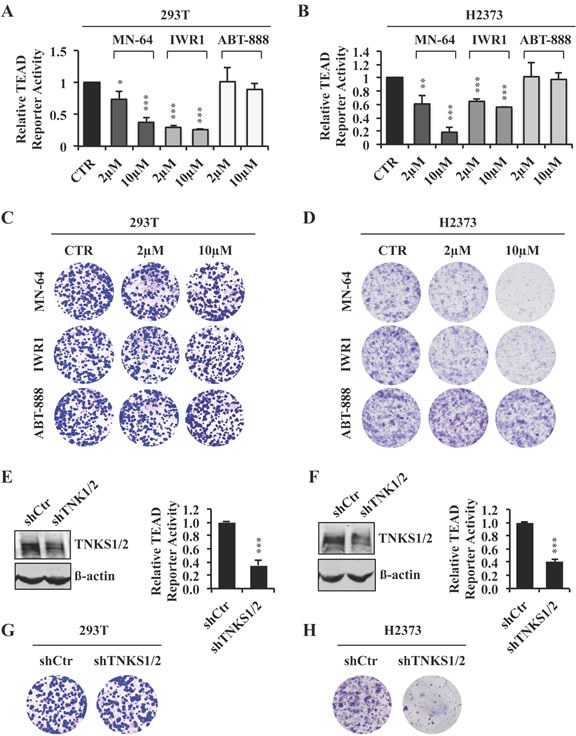
XAV939 downregulates TEAD transcriptional activity through tankyrase inhibition **A.**, **B.** TEAD reporter activity of 293T **A.** or H2373 **B.** cells treated for 24 hours with the indicated inhibitors or CTR. **C.**, **D.** Representative images of colony formation by 293T **C.** or H2373 **D.** cells treated with the indicated inhibitors or CTR. **E.**, **F.** TEAD reporter activity in 293T **E.** or H2373 **F.** cells in the absence or presence of TNKS silencing. Western blot analysis showing knockdown efficiency of TNKS1/2 is also shown. **G.**, **H.** Representative images of colony formation by 293T **G.** or H2373 **H.** cells with TNKS silencing. Error bars indicate SD of representative experiments performed in triplicate. *P≤0.05, **P≤0.01, ***P≤0.001. Student *t-*Test.

XAV939 was initially identified as an inhibitor of both tankyrase 1 and 2 (TNKS1/2), members of the Poly-ADP-ribosyltransferase (PARP) family of enzymes that regulate protein interactions and/or protein stability [25, 26]. To determine whether XAV939's inhibition of TEAD-mediated transcription was indeed the result of TNKS inhibition, we measured TEAD reporter activity in 293T and H2373 cells treated with two other commercially available TNKS inhibitors, MN-64 and IWR1, which each had a different chemical structure [27, 28]. Both compounds were able to inhibit TEAD reporter activity and target gene expression similarly to XAV939 (Figure 3A, 3B and Supplementary Figure S4A–S4F). Furthermore, both MN-64 and IWR1, significantly decreased colony formation by H2373 but not by 293T cells (Figure 3C, 3D). In contrast, treatment with ABT-888, a PARP1/2 specific inhibitor [29], did not affect TEAD reporter activity, target gene expression or cell proliferation under the same conditions (Figure 3A–3D and Supplementary Figure 4SA–4SF).

We also genetically abrogated the expression of endogenous TNKS by lentiviral-mediated transduction of an shRNA that targets TNKS1/2. TNKS1/2 knockdown markedly inhibited TEAD reporter activity, as well as target gene expression, in both 293T and H2373 cells (Figure 3E, 3F and Supplementary Figure S4G, S4H). As with TNK inhibitors, TNKS1/2 silencing inhibited the proliferation of H2373 but not 293T cells (Figure 3G, 3H). All of these results strongly argued that XAV939 functions through TNKS inhibition to specifically downregulate TEAD transcriptional activity and inhibit the proliferation of Hippo mutant tumor cells.

XAV939 was reported to inhibit Wnt signaling by stabilizing Axin and consequently leading to the degradation of ß-catenin [25]. Since the Wnt signaling pathway has recently been implicated in crosstalk with the Hippo pathway [30–33], we investigated the possibility that XAV939 suppressed TEAD transcriptional activity through inhibition of Wnt signaling. Thus, we analyzed Hippo pathway mutant (H2373 and 211H) and non-mutant (293T and MCF10A) cell lines for evidence of upregulated Wnt signaling by means of a TCF luciferase reporter for TCF-ß-catenin-dependent transcription. Whereas HCT116 colon carcinoma cells with Wnt pathway activation by mutant ß-catenin [34] exhibited high TCF reporter activity, the Hippo pathway mutant lines had very low or undetectable TCF reporter activity (Supplementary Figure S5A). These findings excluded the possibility that TEAD transcriptional activity in these lines was inhibited by XAV939 in a Wnt-dependent manner

### TNKS inhibition by XAV939 blocks YAP-dependent transformation through a S127 phosphorylation-independent mechanism

TEAD-mediated transcription is activated by its interaction with the co-transcription factor YAP, whose nuclear localization is highly regulated [1]. LATS1/2-mediated phosphorylation causes YAP to relocalize to the cytosol by a mechanism that involves 14-3-3 binding [21] and targets it for proteasomal degradation as well [35]. YAP activity is also regulated through phosphorylation-independent physical interaction with the angiomotins, a family of proteins that include AMOT, AMOTL1 and AMOTL2. Angiomotin proteins recruit YAP to tight junctions or to the actin cytoskeleton leading to YAP cytoplasmic retention [36].

**Figure 4:**
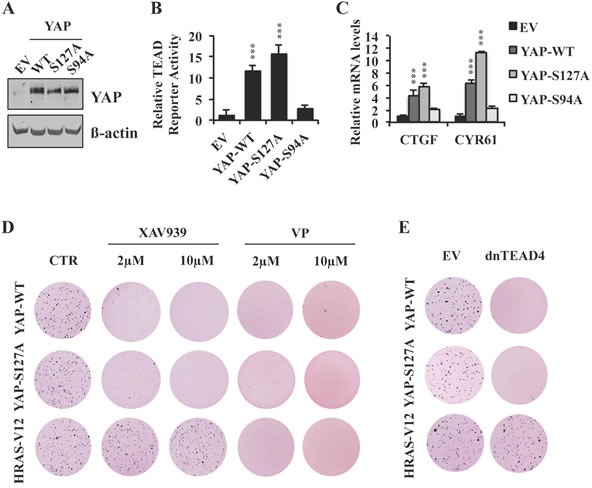
XAV939 inhibits YAP-dependent transformation by a S127 phosphorylation-independent mechanism **A.**-**C.** Western blot analysis **A.**, TEAD reporter activity **B.** and relative mRNA expression of TEAD target genes **C.** in MCF10A cells stably expressing YAP-WT, YAP-S127A or YAP-S94A. **D.** Anchorage-independent growth of MCF10A cells stably expressing YAP-WT, YAP-S127A or HRAS-V12 and treated with XAV939, verteporfin (VP) or CTR. **E.** Anchorage-independent growth of MCF10A cells stably expressing YAP-WT, YAP-S127A or HRAS-V12 in the presence or absence of dnTEAD4 overexpression. Error bars indicate SD of experiments performed in triplicate. ***P≤0.001. Student *t-*Test.

YAP overexpression in MCF10A cells promotes anchorage-independent colony formation in soft agar [37], a property that has been shown to correlate with *in vivo* tumorigenicity [38]. To test the ability of XAV939 to antagonize YAP overexpression by phosphorylation- dependent and independent mechanisms, we stably overexpressed YAP-WT or a YAP-S127A mutant, which has a point mutation in the LATS phosphorylation site required for YAP cytoplasmic retention by 14-3-3 [21]. Both significantly increased TEAD reporter activity and target gene expression, as well as colony formation in soft agar (Figure 4A–4C and Supplementary Figure S5B). In contrast, overexpression of a YAP-S94A mutant, which is unable to bind TEAD [6], failed to induce TEAD transcriptional activity or anchorage-independent growth at similar levels of overexpression (Figure 4A–4C and Supplementary Figure S5B). Of note, XAV939 completely abolished YAP-S127A as well as YAP-WT-induced anchorage-independent cell growth (Figure 4D), consistent with a mechanism of XAV939 action independent of LATS1/2-mediated phosphorylation of YAP-S127.

A recent study indicated that HRAS-V12 overexpression stabilizes YAP protein levels and induces anchorage independent growth by a YAP-dependent mechanism in BJ cells [39]. When we stably overexpressed HRAS-V12 in MCF10A cells, we did not observe any changes in either YAP protein levels or its phosphorylation at S127, whereas the RAS pathway was indeed activated as confirmed by increased levels of pERK (Supplementary Figure S5C). Moreover, TEAD reporter activity was not increased in HRAS-V12 overexpressing compared to vector control MCF10A cells (Supplementary Figure S5D), arguing that the RAS transformed phenotype, including acquisition of agar colony forming ability, was independent of deregulated Hippo transcription in these cells. XAV939 lacked any effect on HRAS-V12-induced colony formation (Figure 4D), results consistent with the specificity of dnTEAD4, which blocked YAP but not RAS induced agar growth (Figure 4E). In striking contrast, verteporfin, an inhibitor that has been reported to interfere with TEAD-YAP protein-protein interactions [40], completely blocked agar colony formation by both YAP and RAS transformed MCF10A cells (Figure 4D). Together, these findings demonstrate that XAV939, but not verteporfin, specifically targets TEAD transcriptional activity and YAP-mediated transformation.

### XAV939 increases YAP cytoplasmic localization independent of S127 phosphorylation

**Figure 5:**
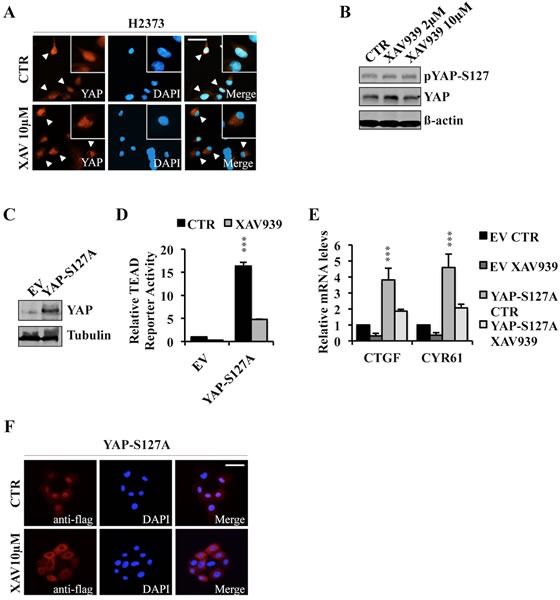
XAV939 induces YAP cytoplasmic relocalization **A.**, **B.** Immunofluorescence images of endogenous YAP expression **A.** and western blot analysis of indicated proteins **B.** in H2373 cells treated with XAV939 or CTR for 24 hours. **C.**-**E.** Western blot analysis **C.**, TEAD reporter activity **D.** and relative mRNA expression levels of TEAD target genes **E.** in MCF10A cells stably overexpressing YAP-S127A. **F.** Immunofluorescence analysis of MCF10A stably expressing YAP-S127A treated with XAV939 or CTR for 24 hours. Bar: 10 μm. Error bars represent SD of experiments performed in triplicate. ***P≤0.001. Student *t-*Test.

To further investigate XAV939's mechanism of action, we analyzed YAP sub-cellular localization in the presence or absence of XAV939. Immunofluorescence staining demonstrated that YAP was mainly localized in the nucleus of untreated NF2 mutant H2373 cells, whereas XAV939 treatment induced YAP re-localization to the cytoplasm of these same cells (Figure 5A). Moreover, H2373 cells treated with varying XAV939 concentrations did not show any significant differences in YAPS127 phosphorylation status as assessed by Western blot (Figure 5B). We next tested the effects of XAV939 on TEAD transcriptional activity and subcellular localization of YAP-S127A in MCF10A cells. Both TEAD reporter activity and expression of target genes were inhibited by XAV939 treatment (Figure 5C–5E). Furthermore, this decrease was associated with a significant shift of YAPS127A to the cytoplasm (Figure 5F). All of these results indicated that XAV939 inhibited TEAD transcriptional activity by a mechanism involving YAP cytosolic re-localization independent of S127 phosphorylation, excluding a LATS-dependent mechanism of YAP sequestration by 14-3-3 and potentially implicating angiomotins.

### TNKS inhibition downregulates YAP activity by stabilizing angiomotins

TNKS catalyze the covalent linkage of ADP-ribose polymer chains to target proteins, regulating their ubiquitylation, stability, and function [41]. It was previously reported that AMOT is degraded by the proteasome [42]. Moreover, *in silico* analysis revealed that all three angiomotin family members contain a recently identified consensus sequence for TNKS substrates [41] and that this consensus sequence is evolutionary conserved (Supplementary table S1). Thus, we hypothesized that XAV939 might act to stabilize angiomotins by inhibiting their tankyrase-mediated degradation.

**Figure 6:**
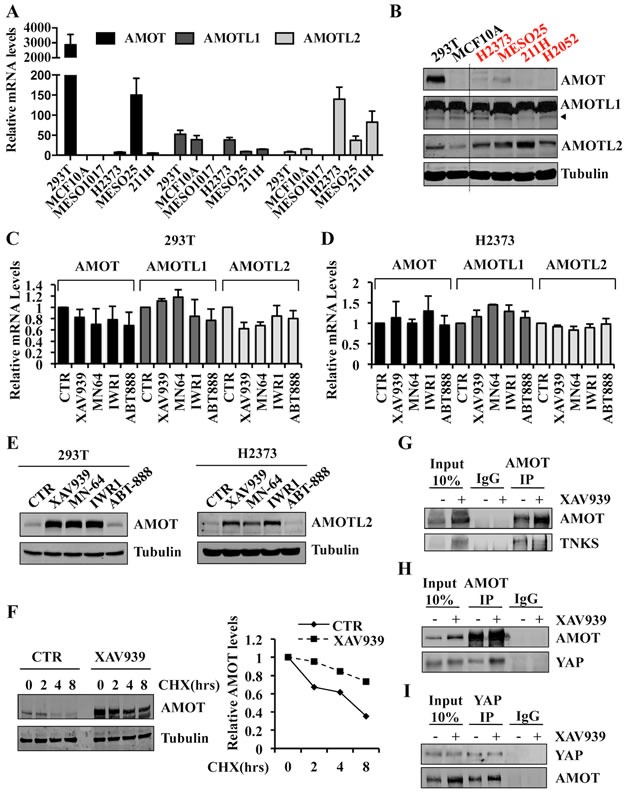
Tankyrase inhibition stabilizes angiomotin proteins and increases AMOT-YAP protein complex formation **A.** mRNA expression levels of AMOT, AMOTL1 and AMOTL2 in the indicated cell lines. Values are represented relative to AMOT levels in MCF10A. **B.** Western blot analysis showing AMOT, AMOTL1 and AMOTL2 expression in the indicated cell lines. **C.**, **D.** mRNA expression levels of AMOT, AMOTL1 and AMOTL2 in 293T **C.** and H2373 **D.** cells treated with 10 μM of the indicated inhibitors or CTR for 24 hours. **E.** Western blot analysis of 293T and H2373 cells treated as in **C.**, **D. F.** Western blot analysis of 293T cells treated with 10μM of XAV939 or CTR for 24 hours. At 24 hours, cycloheximide (20 μg/ml) was added for additional times as indicated. AMOT and Tubulin protein levels were quantified with an Odyssey Infrared Imaging System, and relative expression levels are as shown. **G.** Co-immunoprecipitation of endogenous AMOT and TNKS in 293T CTR cells or treated with 10μM of XAV939 for 24 hours. **H.** Co-immunoprecipitation of endogenous AMOT and YAP in 293T CTR cells or treated with 10μM of XAV939 for 24 hours. **I.** Co-immunoprecipitation of endogenous YAP and AMOT in 293T CTR cells or treated with 10μM of XAV939 for 24 hours. In all co-immunoprecipitation experiments, 10% of total cell lysate was used as Input. Error bars indicate SD of experiments performed in triplicate.

By qRT-PCR and Western blot analyses, we found that expression levels of the three-angiomotin genes varied in Hippo pathway mutant and wild-type cell lines (Figure 6A, 6B). In both 293T and H2373 cells, XAV939, MN-64 or IWR1 treatment did not markedly affect AMOT, AMOTL1, or AMOTL2 mRNA levels (Figure 6C, 6D), but strikingly increased angiomotin protein levels, as shown for AMOT and AMOTL2, respectively (Figure 6E). In contrast, the PARP inhibitor, ABT-888, lacked any effect on either mRNA or protein expression of these same genes (Figure 6C–6E). We also observed increased AMOTL2 levels in Hippo mutant 211H and MESO25 cells upon XAV939 treatment (Supplementary Figure S5E, S5F). These results indicated that TNKS inhibition either increased AMOT protein translation or stabilization. Cycloheximide chase experiments demonstrated increased half-life of endogenous AMOT in the presence of XAV939 (Figure 6F), strongly arguing for a mechanism involving AMOT protein stabilization.

We next investigated the ability of AMOT and TNKS to form an endogenous complex and observed that anti-AMOT co-immunoprecipitated TNKS (Figure 6G). Increased TNKS protein levels were also detected in cell lysates in response to XAV939 treatment, consistent with stabilization of TNKS due to XAV939 inhibiting its autoparsylation and proteasome degradation [43, 44]. Despite higher TNKS protein levels, we detected reduced AMOT-TNKS complex formation in the presence of XAV939 (Figure 6G). Finally, co-immunoprecipitation of endogenous AMOT or YAP in the presence or absence of XAV939 treatment revealed an enrichment of the AMOT-YAP protein complex in treated cells (Figure 6H, 6I). Our findings that XAV939 treatment results in increased YAP sequestration by AMOT as well as YAP cytoplasmic re-localization establish that TNKS inhibitors antagonize YAP-dependent TEAD transcriptional activity.

### Angiomotin stabilization by XAV939 determines its ability to inhibit Hippo mutant tumor proliferation

**Figure 7:**
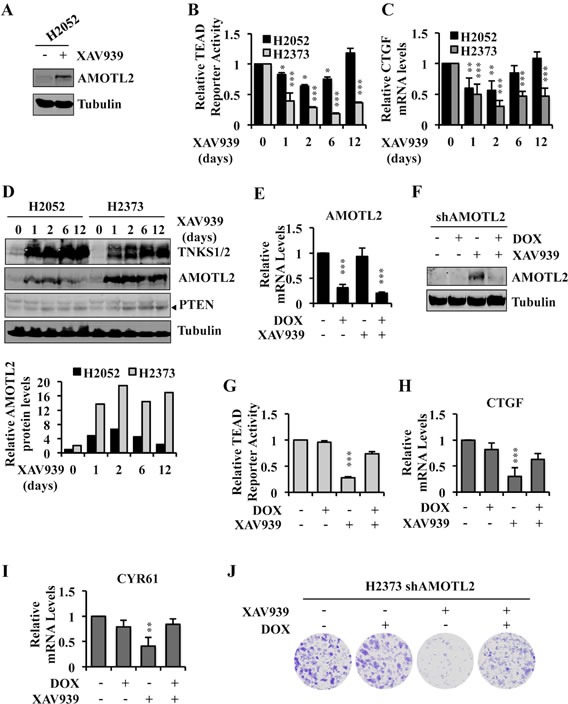
Angiomotin stabilization determines the ability of XAV939 to inhibit TEAD-mediated transcription and proliferation of Hippo pathway mutant tumor cells **A.** Western blot analysis of H2052 cell lysates following treatment with 10μM of XAV939 or CTR for 24 hours. **B.** TEAD reporter activity in H2052 and H2373 cells treated with 10μM of XAV939 or CTR for the indicated time points. Fresh medium with XAV939 was replaced every 2 days. **C.**, **D.** Relative CTGF mRNA expression level **C.** and western blot analysis of TNKS, AMOTL2 and PTEN **D.** in H2052 and H2373 treated as in B. AMOTL2 and Tubulin protein levels in **D.** were measured with the Odyssey Infrared Imaging System and relative expression normalized to H2052 *t* = 0 as shown. **E.**, **F.** Relative AMOTL2 mRNA expression and protein level in H2373 cells stably expressing doxycycline-inducible shRNA, treated with 1μg/ml of doxycycline (DOX) for 72 hours and with 10μM of XAV939 or CTR in the 24 hours prior to lysing the cells. **G.**-**I.** TEAD reporter activity **G.** and relative mRNA expression levels of TEAD target genes **H.**, **I.** in H2373 cells treated as in E. **J.** Representative images of colony formation by H2373 cells treated initially as in E and then cultured under the same conditions for a total of 14 days by replacing the media containing XAV939 or DOX, as indicated, every 48 hours. Error bars indicate SD of experiments performed in triplicate. *P≤0.05, **P≤0.01, ***P≤0.001. Student *t-*Test.

H2052 cells were exquisitely sensitive to dnTEAD4 inhibition of TEAD transcriptional activity and proliferation (Figure 1) but resistant to XAV939 (Figure 2). While XAV939 treatment resulted in increased AMOTL2 protein levels in H2052 cells at 24 hrs (Figure 7A and Supplementary Figure S6A), time course experiments revealed that TEAD transcriptional activity was inhibited more strongly and durably in XAV939 sensitive H2373 cells compared to resistant H2052 cells over the 12 days of treatment (Figure 7B, 7C). Similarly, XAV939 treatment stabilized higher, durable levels of AMOTL2 protein in H2373 as compared to resistant H2052 cells (Figure 7D). These differences were not accounted for by differences in AMOTL2 mRNA levels, which were similar in the two lines (Supplementary Figure S6B). TNKS have been reported to parsylate itself as well as several other substrates leading to their ubiquitin-mediated proteasome degradation [45]. To compare the effectiveness of XAV939 in both resistant and sensitive cell lines, we measured TNKS protein levels, which increased upon XAV939 treatment even more in the resistant line (Figure 7D and Supplementary Figure S6C). Levels of PTEN, another reported TNKS substrate, increased modestly in both cell lines under the same conditions (Figure 7D). Further studies will be needed to understand the basis for the lack of durable angiomotin stabilization in the resistant line.

We next sought to genetically establish that the mechanism by which XAV939 inhibited TEAD transcriptional activity was specifically mediated by inhibition of angiomotin degradation. Silencing of AMOTL2 expression in H2373 cells by lentiviral transduction of AMOTL2 shRNA (Figure 7E, 7F) almost completely rescued the inhibitory effects of XAV939 on TEAD transcriptional activity (Figure 7G–7I and Supplementary Figure S6D) as well as on TEAD-mediated cell proliferation, as assayed by colony formation (Figure 7J). All of these results argue that the growth inhibitory effects of XAV939 in Hippo pathway mutant tumor cells were primarily due to its inhibition of TNKS-mediated angiomotin degradation.

## DISCUSSION

Our present studies establish that human tumor lines harboring mutations in Hippo pathway core components, LATS or NF2, exhibited constitutively up-regulated TEAD transcriptional activity compared to Hippo wild-type cells, whose low levels of transcription were regulated by both serum and cell density. We also observed much higher YAP protein levels in Hippo pathway mutant compared to wild-type cells, consistent with evidence that NF2 and LATS regulate YAP activity and protein stability [21, 35, 46]. In contrast to Hippo wild-type cells whose TEAD-mediated transcription appeared to be dispensable for proliferation, Hippo pathway mutant tumor cells exhibited striking inhibition of proliferation in response to down regulation of TEAD transcriptional activity. These findings provide strong evidence for the critical importance of constitutively up-regulated TEAD-mediated transcription for Hippo pathway mutant tumor cells. While the mechanisms involved in this dependency remain to be elucidated, our results argue that agents that specifically target the constitutively high TEAD transcriptional activity in Hippo pathway deregulated tumors should exhibit a high therapeutic index in targeting such tumors.

We included the TNKS inhibitor, XAV939, in a screen for small molecule inhibitors of TEAD transcriptional activity based on reports of Wnt/Hippo pathway crosstalk [30–32] and evidence that XAV939 antagonizes TNKS parsylation-mediated degradation of Axin to inhibit canonical Wnt signaling [25]. Having identified XAV939 in this screen, we showed that it as well as other TNKS inhibitors and TNKS1/2 knockdown inhibited TEAD-mediated transcription, whereas an inhibitor of related members of the PARP superfamily lacked this activity. XAV939 phenocopied the effects of dnTEAD4 in inhibiting TEAD transcriptional activity and inducing a G1 growth arrest in most of the LATS or NF2 mutant tumor lines analyzed without detectable growth inhibitory effects on other cells tested. It was possible to exclude involvement of the canonical Wnt pathway, since none of the Hippo pathway mutant lines analyzed exhibited increased TCF reporter activity, a sensitive marker of Wnt pathway activation [47].

Mechanistic studies revealed that XAV939 treatment did not affect YAP phosphorylation and resulted in cytoplasmic retention of YAP independent of YAP phosphorylation on S127, required for YAP cytoplasmic sequestration by 14-3-3 [21]. Angiomotins, which sequester YAP independent of phosphorylation [36], possess a recently identified highly conserved consensus sequence for TNKS substrates [41], and TNKS inhibition increased angiomotin family protein expression by a mechanism involving protein stabilization. Moreover, increased angiomotin levels in response to XAV939 resulted in increased YAP complex formation with angiomotin, known to sequester YAP in the cytosol [36]. TNKS have been reported to influence other processes involved in growth control in addition to Wnt signaling including regulation of telomere length (TRF1), spindle polarity (NUMA), DNA repair (DNAPK), metabolism (GLUT4) and tumor suppression (PTEN) through paryslation-mediated degradation or stabilization [45, 48]. We showed that knockdown of AMOTL2, the predominant angiomotin family member expressed in Hippo pathway mutant H2373 cells, almost completely rescued these cells from XAV939 inhibition of TEAD-mediated transcription and proliferation. All of these findings establish that TNKS inhibitors antagonize Hippo pathway mutant tumor cells primarily through angiomotin stabilization independent of other TNKS functions.

A small molecule inhibitor, verteporfin, and a polypeptide termed super-TDU, comprising the TEAD binding domain of VGLL4, a TEAD transcriptional repressor [49], have been reported to physically interfere with TEAD-YAP interactions and to antagonize TEAD transcriptional activity [40, 50]. Verteporfin suppressed liver tumor growth induced by YAP overexpression or NF2 inactivation in mice [40], and super-TDU suppressed growth of gastric tumor xenografts with Hippo pathway deregulation [50]. While it is not yet known the degree to which super-TDU may be specific for Hippo pathway deregulated tumor cells, we found that verteporfin blocked anchorage-independent growth of RAS transformed cells, which was not inhibited by either dnTEAD4 or XAV939. These results argue against verteporfin's Hippo pathway specific actions. In line with our findings, a recent publication showed a YAP-independent tumor suppressive function of verteporfin in colorectal cancer [51].

While our manuscript was in preparation, Wang et al. reported identification of XAV939 in a screen for small molecule inhibitors of TEAD transcriptional activity [52]. They showed that XAV939 stabilized angiomotin and inhibited acini formation in matrigel by YAP overexpressing MCF10A cells [52]. They also reported that the E3 ligase, RNF146, previously identified to work in concert with TNKS to target parslyated proteins such as Axin and PTEN for proteasome-mediated degradation [44, 48], was the E3 ligase responsible for TNKS-mediated angiomotin degradation [52]. There is previous evidence that angiomotins have tumor suppressive functions by sequestering YAP in the cytosol and by causing cellular transformation when depleted in immortalized MDCK and MCF10A cells [36, 53]. However, there is also a report showing that angiomotins can play a positive role in YAP-mediated cell proliferation in the liver [54]. Wang et al and our independent findings provide strong complementary evidence that the mechanism of XAV939 inhibition of TEAD transcriptional activity involves angiomotin stabilization. Moreover, our studies directly establish the biological importance of this mechanism in specifically targeting the proliferation of human tumor cells with mutations in Hippo pathway core components.

Among tumor lines with Hippo pathway mutations analyzed by us, one mesothelioma, H2052, with both LATS2 and NF2 mutations, was found to be resistant to XAV939 despite its striking sensitivity to dnTEAD4 inhibition of TEAD-mediated transcription and proliferation. AMOTL2, the most abundant angiomotin in both resistant H2052 and sensitive H2373 tumor cells, showed lower and less durable stabilization in H2052 cells in response to XAV939. One possible explanation could be that another ubiquitin ligase(s) acts independently of TNKS, to preferentially inhibit angiomotin accumulation in the resistant tumor cells. However, mechanistic understanding, as well as the frequency of the recurrence of such resistance and the effectiveness of TNKS inhibitors in tumors with other Hippo pathway lesions, awaits further studies. Nonetheless, our findings indicate that the level of angiomotin protein stabilization could potentially provide a useful biomarker with which to assess the sensitivity of Hippo pathway mutant tumors to TNKS inhibitors.

Our findings that TNKS inhibitors predominately induced G1 arrest rather than cell death in Hippo pathway mutant tumor cells have potential parallels with the G1 arrest induced by tyrosine kinase pathway inhibitors in solid tumor cells [55, 56]. Several studies revealed that growth factor signaling pathways also activate pro-survival signaling and can be used in cooperation with standard chemo/irradiation therapies [57, 58]. While there is some evidence suggesting that TEAD-YAP transcription may have pro-survival properties [59, 60], further studies will be needed to determine whether inhibition of TEAD-YAP signaling can cooperate with chemo/irradiation therapies.

Under physiological conditions, growth factor signaling pathways are subject to stringent regulation through negative feedback mechanisms, which limit the strength and duration of such signaling. The development of biologically targeted therapies for oncogene activated signaling has revealed that pathway inhibition can relieve negative feedback, which can then promote oncogenic signals and contribute to therapy resistance. For example, a recent screen for genes increasing the efficacy of RAF inhibitors in cancer cells harboring BRAF-V600E mutations identified YAP as a key to drug resistance, and combined YAP and RAF or MEK inhibition was found to be synthetically lethal for BRAF and RAS mutant tumors [61]. Thus, it will be of interest to determine the extent to which TNKS inhibitors cooperate with RAF or MEK inhibitors in targeting such tumors as well as how BRAF or MEK inhibition may cooperate with down regulation of YAP-dependent TEAD transcriptional activity by TNKS inhibitors in Hippo pathway mutant tumors.

Within the PARP superfamily, specific inhibitors of PARP1/2 are now in the clinic [62]. Efforts aimed at developing TNKS inhibitors to target Wnt activated tumors have recently led to new compounds with better drug-like properties compared to XAV939 with evidence of some efficacy in Wnt tumor models [63, 64]. Nonetheless, stability issues, dose-limiting toxicity and weight loss attributed to Wnt inhibitory effects in the gastrointestinal tract [63, 64] pose challenges to their application as therapeutic agents. Thus, TNKS inhibitors with improved drug-like properties and/or less toxicity will likely be needed. However, the refractory nature of tumors such as mesothelioma to current treatments and the identification of angiomotin, whose stabilization by TNKS inhibitors specifically antagonizes the proliferation of such tumor cells, argues that approaches aimed at angiomotin stabilization could eventually lead to new targeted therapies for the increasing array of Hippo pathway deregulated tumors for which there are as yet no effective therapies.

## MATERIALS AND METHODS

### Cell culture and treatments

293 (CRL-1573), 293T (CRL-3216), MCF10A (CRL-10317), H2052 (CRL-5915), 211H (CRL-2081), H2373 (CRL-5943) were obtained from ATCC. MESO25 was a gift from J. Testa (Fox Chase Cancer Center, Philadelphia, PA, USA). 293 and 293T cells were cultured in Dulbecco's Modified Eagle's Medium (DMEM) (Invitrogen, Carlsbad, CA) supplemented with 10% Fetal Bovine Serum (FBS) (Sigma-Aldrich, St. Louis, MO), 50 units/ml of penicillin/streptomycin. H2373, MESO25, MSTO-211H (211H) and H2052 cells were cultured in RPMI-1640 medium supplemented with 10% FBS and 50 units/ml of penicillin/streptomycin. MCF10A cells were grown in DMEM/F12 medium supplemented with 5% horse serum, 10μg/ml insulin, 100ng/ml cholera toxin, 0.5mg/ml hydrocortisone, 20ng/ml EGF and 50 units/ml of penicillin/streptomycin. Cells were cultured at 37°C and 90% humidity in a 5% CO_2_ incubator. Cycloheximide was purchased from Sigma (Saint Louis, MO, USA). The following inhibitors were used: XAV939 (Maybridge, #03920SC), MN-64 (Sigma, #SML1012), IWR1 (Sigma, #I0161), PARP1/2 inhibitor, ABT-888 (Veliparib, Selleck Chemicals, #S1004); verteporfin (Sigma, # SML:0534-5MG). Each inhibitor was dissolved in DMSO and was used at the indicated concentration in medium including 0.1% DMSO. In all experiments, 0.1% DMSO in medium was used as control. Selectable markers to generate stably transduced cells were used as followes: 2μg/ml puromycin (Calbiochem, San Diego, CA, USA), 400 μg/ml hygromycin B (Invitrogen, Carlsbad, CA, USA), 1μg/ml doxycycline (Sigma, Saint Louis, MO, USA).

### Plasmids and viral infections

A TEAD reporter was generated by cloning 10 copies of GT-IIC motif (GTGGAATGT) into a NV-Luciferase vector [65] using ClaI and NheI restriction sites. pQCXIH-Myc-YAP, pQCXIH-Flag-YAP-S127A and pQCXIH-Myc-S94A were purchased from Addgene (Plasmid #33091, #33092 and #33094). The pQCXIH vector control was generated by removing YAP and religating the vector backbone. pBABE-puro and pBabe-puro-HRAS-V12 vectors were previously described [66]. dnTEAD4 was cloned from the pSPORT6 Vector (Dharmacon, Lafayette, CO, USA) into NSPI-CMV-MCS lentiviral vector [67] using the following primers containing Nhe1 and BamH1 restriction sites: FW-TAAGCAGCTAGCGCCACCTTGGAGGGCACGGCCGGCAC and Rev- ACTATGGGATCCTCA TTCTTTCACCAGCCTGTGGATGTGGTGCTGAGC. The dominant negative (dn) mutation, Y429H (TAC—> CAC) [6], was introduced into TEAD4 gene by site-directed mutagenesis. We generated stable shRNA and inducible shRNA vectors by cloning the oligos into pLKO.1 or pLKO-Tet-Puro vectors, respectively. The sequences of the specific oligos used in the study will be provided upon request. Retro and lenti-virus production and infection were carried out as previously described [67].

### Small-molecule inhibitor screen and reporter luciferase assay

A compound library consisting of 277 novel kinase inhibitors [68] and few commercially available inhibitors were used to screen for effects on the TEAD luciferase reporter assay. 293 cells expressing the TEAD reporter along with firefly-renilla luciferase (20:1 ratio) were plated at low density (2×10^4^ cells) in 24 well plates in triplicate. 24 hours after plating, the cells were treated with 10μM of each compound or DMSO as control. 24 hours later, dual-luciferase reporter assay was performed according to the manufacturer's protocol (Promega, Madison WI, USA), using TD-20e Luminometer (Turner Biosystem, Promega, Madison WI, USA). TEAD reporter activity was normalized to renilla luciferase. The Log2 values were calculated for each compound using the DMSO sample as control. Potential hits were repeated in both 293 and 293T cells with similar results.

### Cell proliferation assay

For clonogenic proliferation assay, cells were plated in triplicate at 1×10^3^ cells in 6-well plates. For analysis of the effects of inhibitors on cell proliferation, fresh medium with inhibitor was replaced every 48 hours. After 10 to 14 days of treatment, cultures were fixed and stained with 1% crystal violet (in ethanol) and photographed.

### Anchorage-independent growth assay

For analysis of anchorage-independent growth, 2.5×10^3^ MCF10A or MCF10A cells stably expressing lentiviral or retroviral transduced cDNAs as indicated were seeded in triplicate in 1ml of growth media containing 0.3% agar (BD #214050) on top of 1ml of 0.48% agar in 35mm dishes. Cells were fed every 4 days for 3 weeks by adding 0.2 mL of growth medium containing either 0.1% DMSO as a control or compounds in 0.1% DMSO at the concentrations indicated. Colonies were then fixed and stained with 1% crystal violet (in ethanol) and photographed.

### mRNA extraction and cDNA synthesis

Total RNA was extracted from cells using the RNeasy Mini kit (Qiagen, Hilden, Germany) following the manufacturer's instructions. 1μg of total RNA was used for cDNA synthesis using Superscript II (Invitrogen, Carlsbad, CA, USA) according to the manufacturer's instructions.

### Quantitative real-time PCR analysis

Quantitative RT-PCR was performed using the ViiA™ 7 Real-Time PCR System (Life Technologies, Carlsbad, CA, USA) using the FastStart SYBR Green Master mix (Roche, Indianapolis, IN, USA). Primers were as follows: CTGF FW-CCAATGACAACGCCTCCTG, Rev-TGGTGCAGCCAGAAAGCTC; CYR61 FW- AGCCTCGCATCCTATACAACC, Rev- TTCTTTCACAAGGCGGCACTC; ANKRD1 FW- CACTTCTAGCCCACCCTGTGA, Rev- CCACAGGTTCCGTAATGATTT; YAP FW-TAGCCCTGCGTAGCCAGTTA, Rev TCATGCTTAGTCCACTGTCTGT, AMOT FW-ACTACCACCACCTCCAGTCA, Rev-ACAAGGTGACGACTCTCTGC; AMOTL1 FW-GCAGACAGGAAAACTGAGGA, REV-AAATGTGGTGGGAACAGAGA; AMOTL2 FW-GCTACTGGGGTAGCAACTGA, Rev-GAAGGCAGTGAGGAACTGAA; TNKS1 FW-GACCCAAACATTCGGAACAC, Rev-GCAGCTTCTAGGAGTTCGTCTT; TNKS2 FW-AACGAGTCAAGAGGCTGGTG, REV-TTCAACTACGTCTTTCCGCC; GAPDH FW- CTCTGCTCCTCCTGTTCGAC Rev- TTAAAAGCAGCCCTGGTGAC. PCR was performed in 384 well plates in 10 μl total volumes under the following conditions: 95°C for 15 min, followed by 40 cycles of 94°C for 15 sec, 61°C for 30 sec, and 72°C for 30 sec. Specificity was verified by a dissociation curve. Results were analyzed with ViiA7 RUO software (Life Technologies, Carlsbad, CA, USA). Gene expression levels were normalized to GAPDH expression.

### Western blot analysis

Cells were harvested in EBC lysis buffer (50 mM Tris-HCl at pH 7.5, 150 mM NaCl, 5 mM EDTA, 0.5% NP-40), supplemented with Complete Mini Protease and Phosphatase Inhibitor Cocktails (Roche, Indianapolis, IN, USA). Cells were lysed and 30-80 μg protein subjected to SDS-PAGE followed by transfer onto an Immobilon-FL PVDF membrane (Millipore, Billerica, MA, USA) and incubation with the indicated antibodies. Detection was carried out with an Odyssey Infrared Imaging System (LI-COR Biosciences, Lincoln, NE, USA) with IR dye-tagged secondary antibodies (LI-COR Biosciences). The following antibodies were utilized: mouse anti-YAP, goat anti-NF2, mouse anti-AMOT, goat anti-AMOTL1, goat anti-AMOTL2 (Santa Cruz, Dallas, TX, USA), mouse anti-FlagM2 (Sigma, Saint Louis, MO, USA), rabbit anti-LATS1, rabbit anti-LATS2, rabbit anti-p-YAP (Cell Signaling, Danvers, MA, USA), TNKS1/2 (Santa Cruz, Dallas, TX, USA), mouse anti-TEAD4, mouse anti-RAS (Thermo Scientific, Waltham, MA, USA), mouse anti-α-Tubulin, mouse anti-β-actin (Sigma, Saint Louis, MO, USA).

### Immunoprecipitation analysis

Cells were harvested in RIPA lysis buffer (50mM Tris-Cl; pH 8.0, 5mM EDTA, 1% Triton X-100, 0.1% sodium deoxycholate, 0.1% SDS, 150mM NaCl) supplemented with Complete Mini Protease and Phosphotase Inhibitor Cocktails. 800μg proteins were incubated with 10μg of antibody overnight at 4°C. Anti-mouse or anti-rabbit IgG (Santa Cruz, Dallas, TX, USA) was used as a negative control. Immunoprecipitated complexes were captured by 2h incubation at 4°C with Dynabeads Protein A/G B (Invitrogen, Carlsbad, CA, USA), followed by three washes in lysis buffer. Immunoprecipitated complexes were eluted by boiling for 5 min with Laemmli buffer (150 mM Tris-Cl; pH 6.8, 20% glycerol, 4% SDS, 0.002% bromophenol blue, 2% 2-mercaptoethanol) with 10% of the total lysates run on the same gel for comparative immunoblot analysis.

### Immunofluorescence microscopy

Cells cultured on glass coverslips were fixed for 10 min with 4% paraformaldehyde in 1xPBS at 37°C and permeabilized for 3 min with 0.02% Triton-X100, following exposure for 1 hour to a blocking solution (PBS containing 5% BSA). Coverslips were then incubated at room temperature with the following primary antibodies: anti-YAP (Santa Cruz, Dallas, TX, USA) and anti-Flag M2 (Sigma, Saint Louis, MO, USA). Corresponding secondary antibodies were Alexa fluor conjugated (Molecular Probes, Eugene, OR, USA). 2μg/ml DAPI was used as a counter stain and was used to label nuclei. Imaging was performed using an Axioplan 2 Imaging System (Zeiss, Oberkochen, Germany).

## SUPPLEMENTARY MATERIALS FIGURES AND TABLES


