# Supplementary material for: Angiomotin stabilization by tankyrase inhibitors antagonizes constitutive TEAD-dependent transcription and proliferation of human tumor cells with Hippo pathway core component mutations
Source: Oncotarget. 2016 Apr 29;7(20):28765–82. doi: 10.18632/oncotarget.9117 (PMC5045355; doi:10.18632/oncotarget.9117)
Supplement: Supplementary file 1 [file oncotarget-07-28765-s001.pdf]

Angiomotin stabilization by tankyrase inhibitors antagonizes constitutive TEAD-dependent transcription and proliferation of human tumor cells with Hippo pathway core component mutations

Supplementary Material

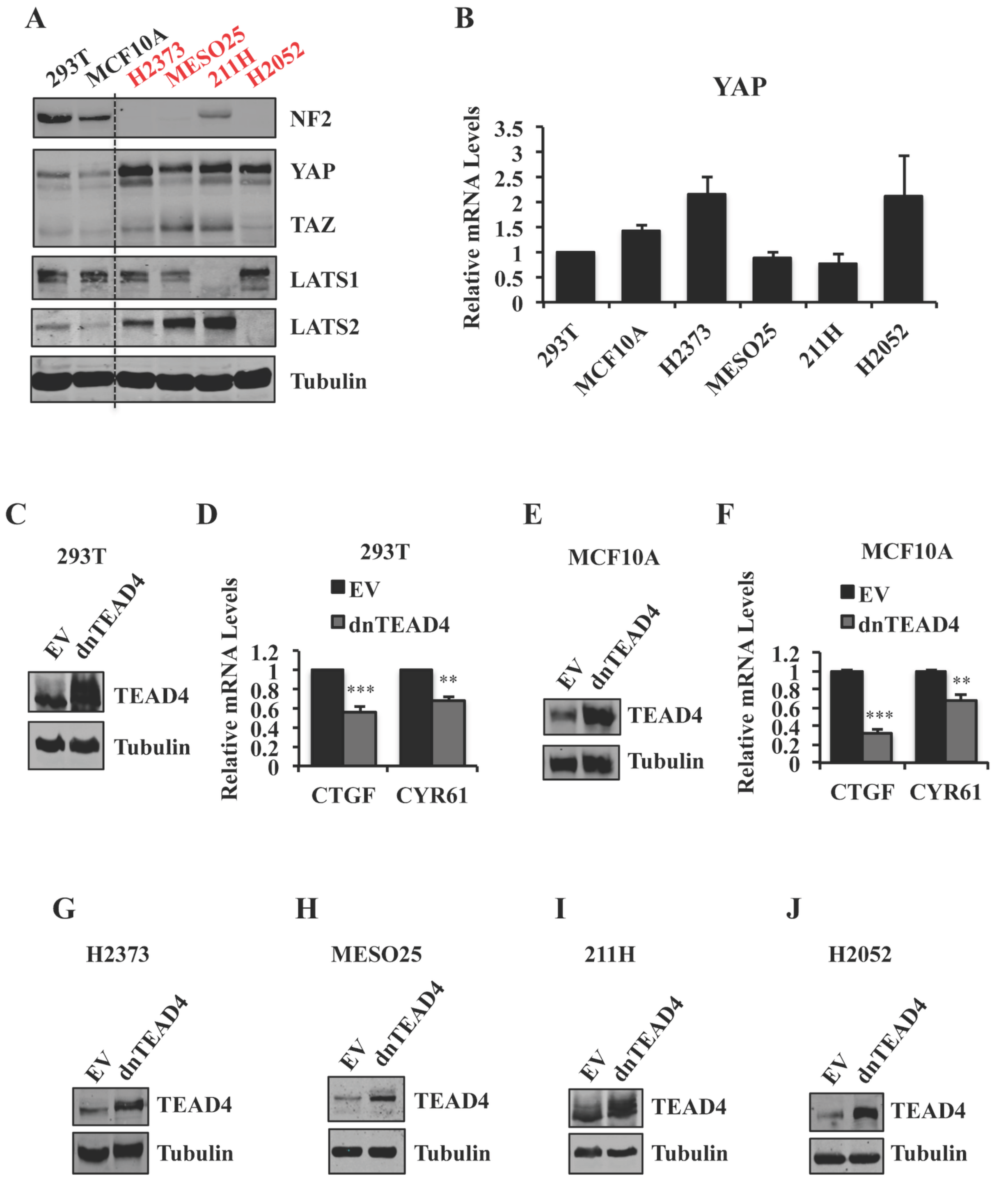

Supplementary Figure S1. dnTEAD4 inhibits TEAD transcriptional activity. (A) Western blot analysis showing the expression of Hippo pathway core components in the indicated cell lines. The discontinuity in the bands is due to deletion of irrelevant lanes in the gel. (B) Relative YAP mRNA expression levels in the indicated cell lines. YAP expression of each line is shown relative to 293T cells. (C) Western blot analysis of 293T cells stably expressing dnTEAD4. (D) Relative CTGF and CYR61 mRNA expression levels in 293T treated as in C. (E) Western blot analysis of MCF10A cells stably expressing dnTEAD4. (F) Relative CTGF and CYR61 mRNA expression levels in MCF10A treated as in E. (G-J) Western blot analysis of indicated cell lines stably expressing dnTEAD4. Error bars indicate SD of experiments performed in triplicate. ***$P \leq 0.001$. Student *t*-Test.

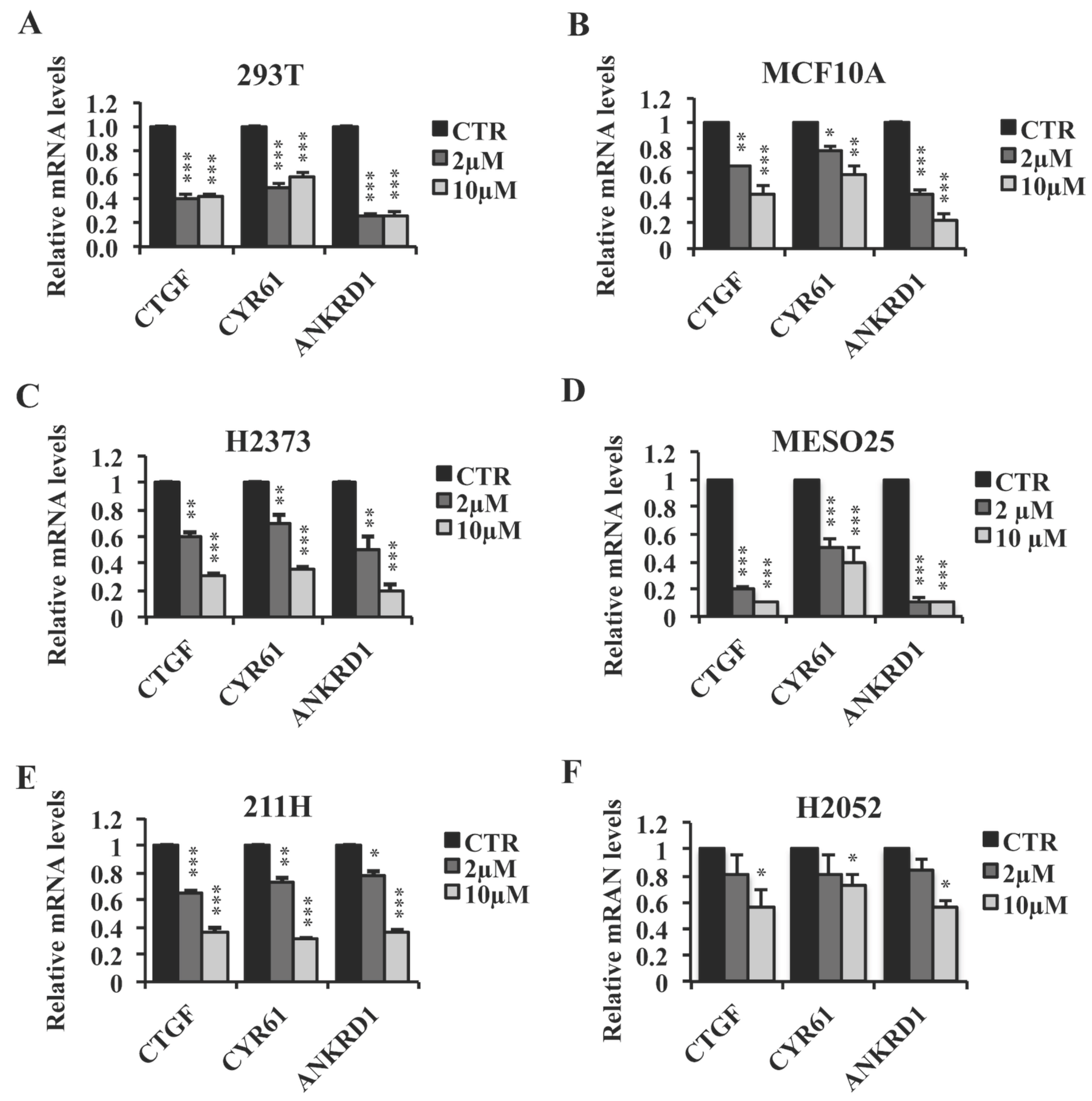

Supplementary Figure S2. XAV939 inhibits TEAD target genes. (A-F) Relative mRNA expression levels of CTGF, CYR61 and ANKRD1 in the indicated cell lines treated with XAV939 or CTR for 24 hours. Error bars indicate SD of experiments performed in triplicate. *P≤0.05, **P≤0.01, ***P≤0.001. Student *t*-Test.

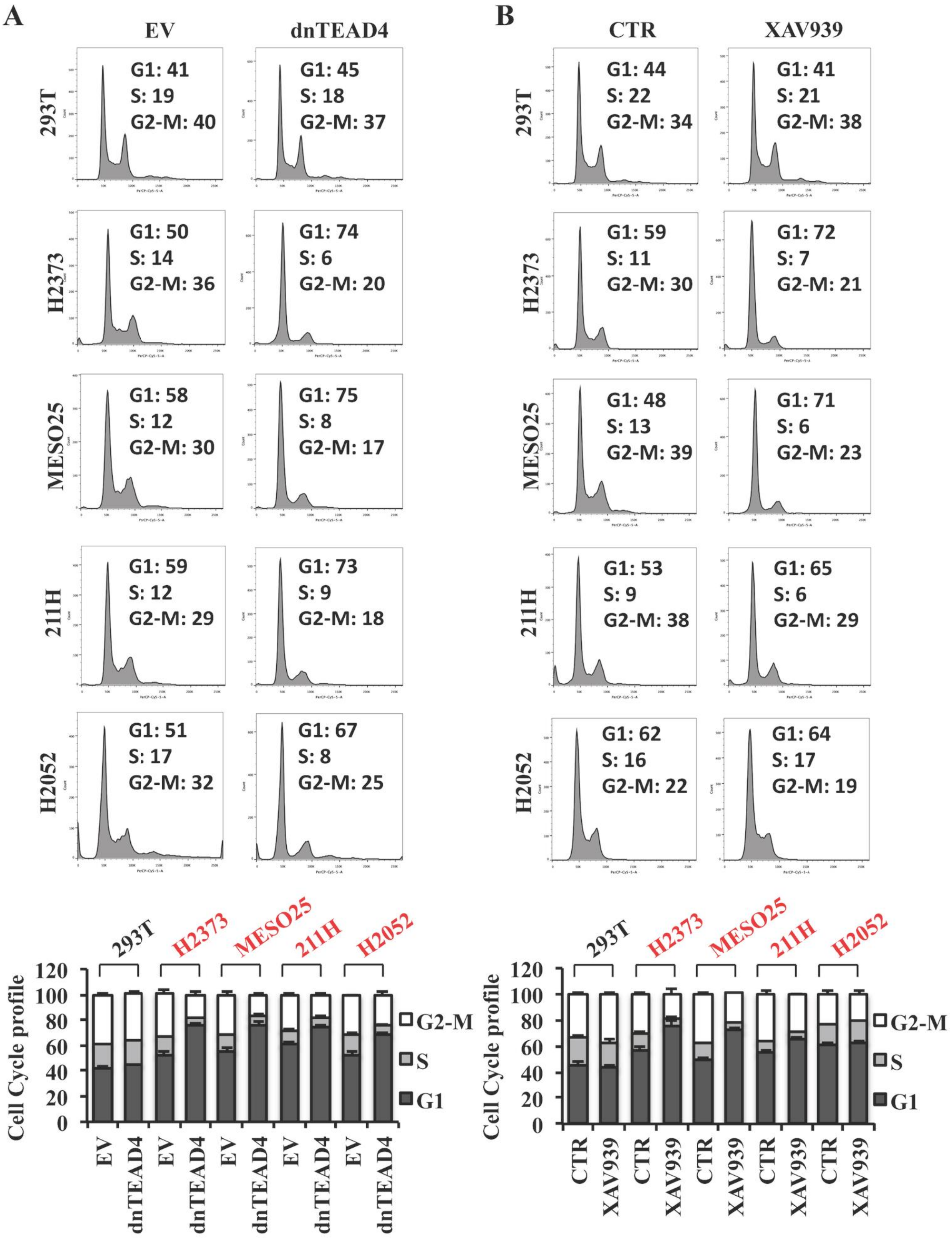
A
EV
dnTEAD4
293T
G1: 41
S: 19
G2-M: 40
G1: 45
S: 18
G2-M: 37
H2373
G1: 50
S: 14
G2-M: 36
G1: 74
S: 6
G2-M: 20
MESO25
G1: 58
S: 12
G2-M: 30
G1: 75
S: 8
G2-M: 17
211H
G1: 59
S: 12
G2-M: 29
G1: 73
S: 9
G2-M: 18
H2052
G1: 51
S: 17
G2-M: 32
G1: 67
S: 8
G2-M: 25
293T
H2373
MESO25
211H
H2052
Cell Cycle profile
120
100
80
60
40
20
0
G2-M
S
G1
EV
dnTEAD4
EV
dnTEAD4
EV
dnTEAD4
EV
dnTEAD4
EV
dnTEAD4
B
CTR
XAV939
293T
G1: 44
S: 22
G2-M: 34
G1: 41
S: 21
G2-M: 38
H2373
G1: 59
S: 11
G2-M: 30
G1: 72
S: 7
G2-M: 21
MESO25
G1: 48
S: 13
G2-M: 39
G1: 71
S: 6
G2-M: 23
211H
G1: 53
S: 9
G2-M: 38
G1: 65
S: 6
G2-M: 29
H2052
G1: 62
S: 16
G2-M: 22
G1: 64
S: 17
G2-M: 19
293T
H2373
MESO25
211H
H2052
Cell Cycle profile
120
100
80
60
40
20
0
G2-M
S
G1
CTR
XAV939
CTR
XAV939
CTR
XAV939
CTR
XAV939
CTR
XAV939

Supplementary Figure S3. dnTEAD4 and XAV939 induce G1 arrest in Hippo pathway mutant tumor cells but not Hippo pathway wild-type cells. (A, B) Cell cycle profile by Propidium Iodide staining of the indicated cell lines stably expressing dnTEAD4 (A) and treated with 10µM of XAV939 or CTR for 72 hours (B). Statistical analysis of the cell cycle profile is shown below. Error bars indicate SD of experiments performed in triplicate.

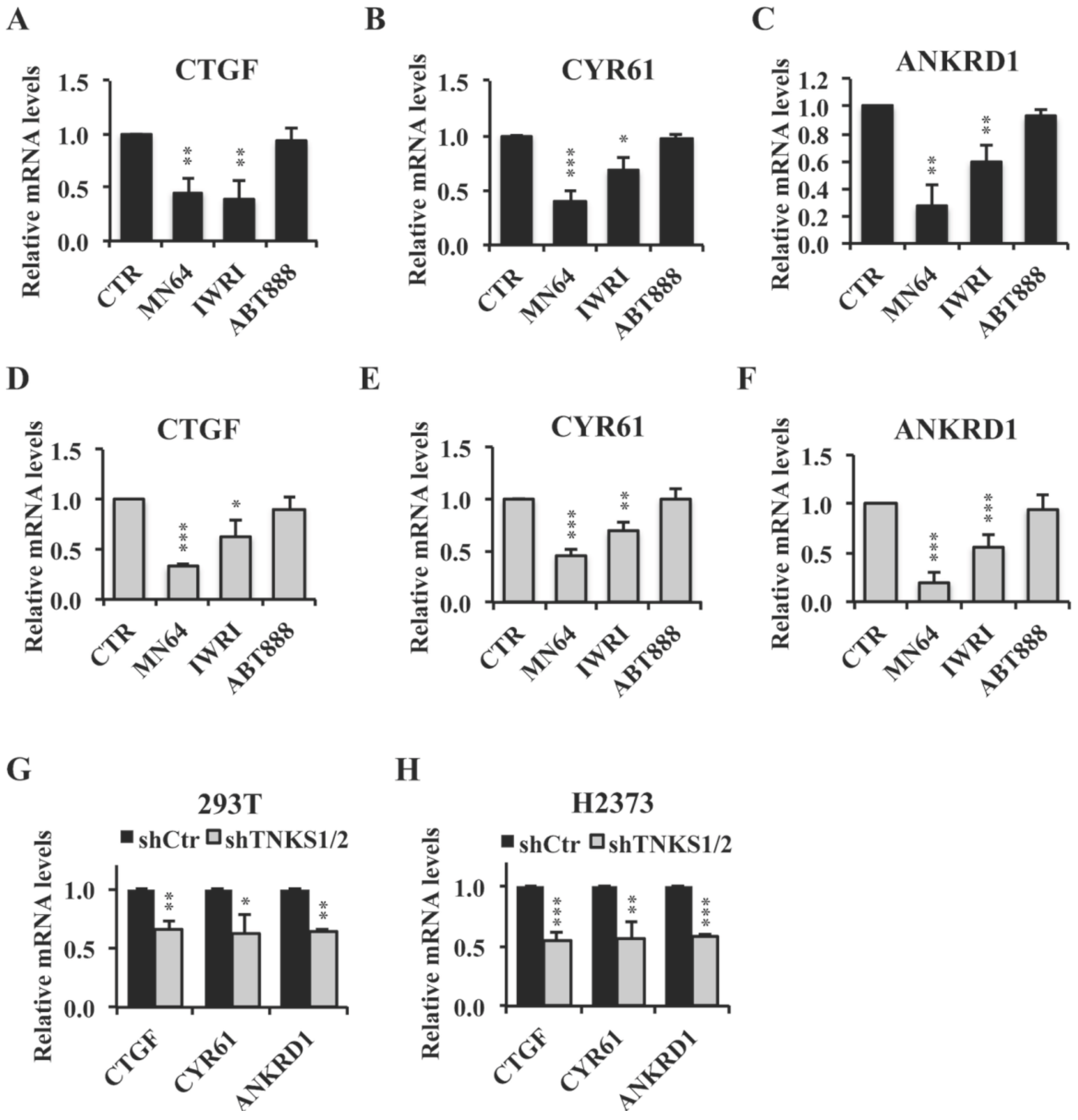

Supplementary Figure S4. Tankyrase inhibition downregulates expression of TEAD target genes. (A-F) Relative mRNA expression levels of CTGF, CYR61 and ANKRD1 in 293T (A-C) and H2373 (D-F) cells treated with 10µM of the indicated inhibitors or CTR for 24 hours. (G, H) Relative mRNA expression levels of CTGF, CYR61 and ANKRD1 in 293T (G) and H2373 (H) cells stably silenced for TNKS1/2. Error bars indicate SD of experiments performed in triplicate. *$P \leq 0.05$, **$P \leq 0.01$, ***$P \leq 0.001$. Student *t*-Test.

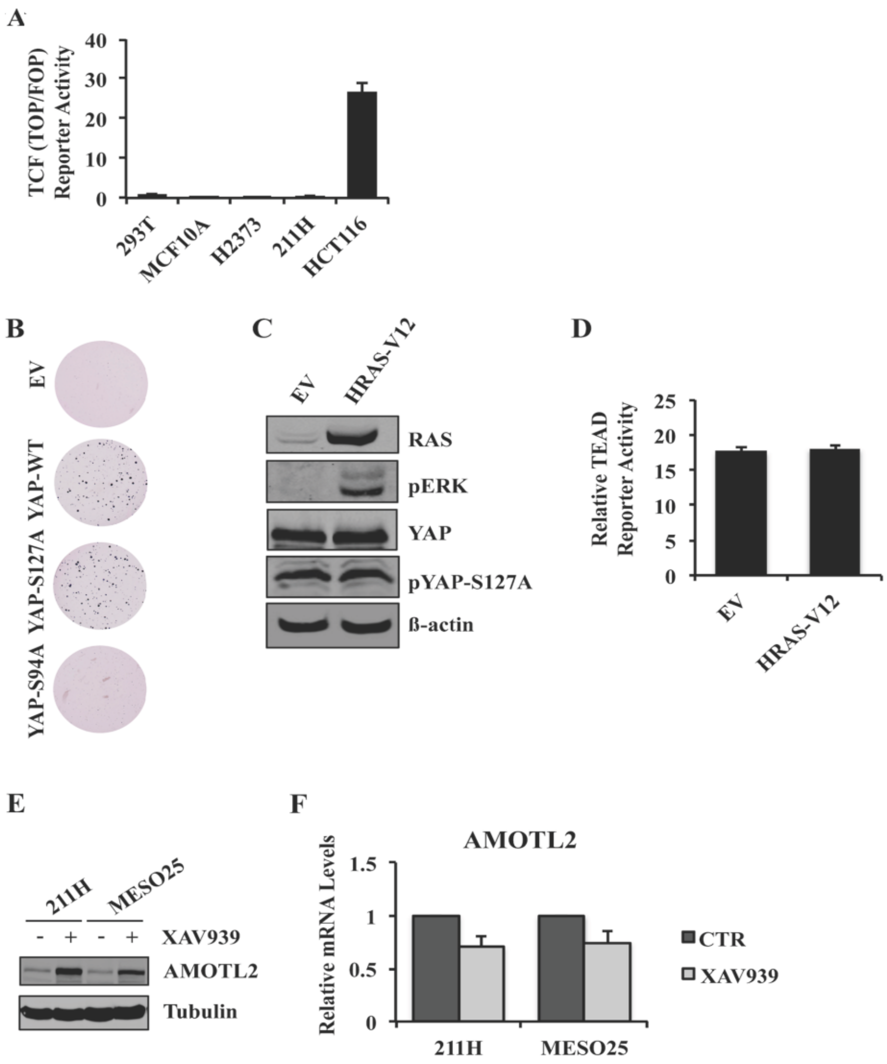

Supplementary Figure S5. (A) TCF (TOP/FOP) reporter activity in the indicated cell lines. (B) Anchorage-independent growth of MCF10A cells stably expressing empty vector (EV), YAP-WT, YAP-S127A or YAP-S94A. (C) Western blot analysis of MCF10A cells stably expressing HRAS-V12 for the proteins indicated. (D) TEAD reporter activity of EV and HRAS-V12 MCF10A cells. (E) Western blot analysis of 211H and MESO25 cells treated with 10μM of XAV939 or CTR for 24 hours. (F) Relative AMOTL2 mRNA expression level in 211H and MESO25 cells treated with 10μM XAV939 or CTR for 24 hours. Error bars indicate SD of experiments performed in triplicate.

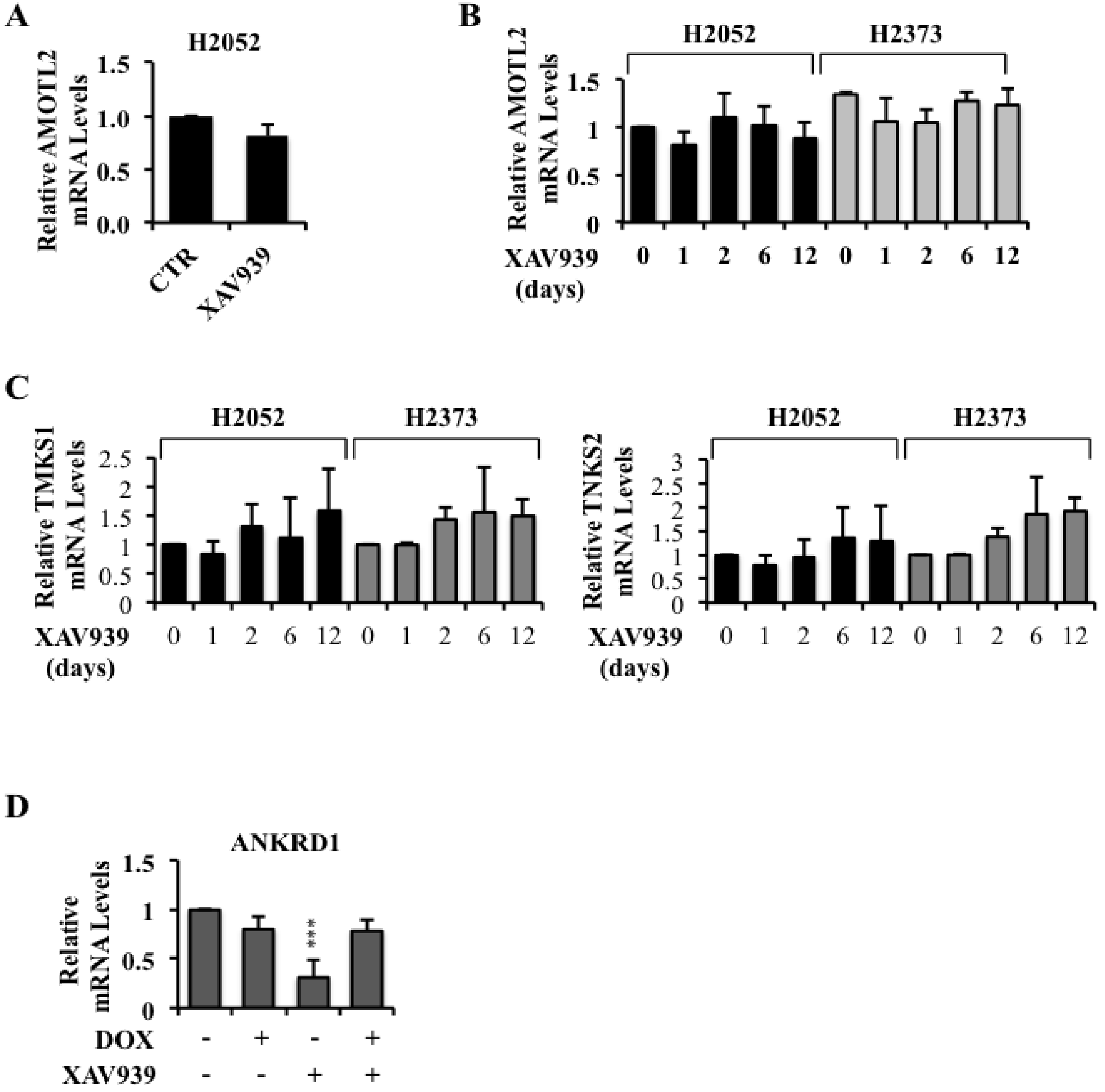

Supplementary Figure S6. AMOTL2 mRNA levels are not affected by XAV939 treatment. **(**A) Relative AMOTL2 mRNA expression level in H2052 cells treated 10μM of XAV939 or CTR for 24 hours. (B, C) Relative AMOTL2, TNKS1 and TNKS2 mRNA expression levels in H2052 and H2373 cells treated with 10μM of XAV939 or CTR for the indicated time points. Values are shown relative to H2052 expression level at time 0. (D) Relative ANKRD1 mRNA expression levels in H2373 cells stably expressing doxycycline-inducible shRNA, treated with

1μg/ml of doxycycline (DOX) for 72 hours and with 10μM of XAV939 or CTR in the 24 hours prior to lysing the cells for RNA extraction. Error bars indicate SD of experiments performed in triplicate.

Supplementary Table S1. A tankyrase binding domain motif [40] depicted in red is evolutionarily conserved in angiomotin family proteins. Cross-species sequences were acquired through the UCSC genome browser (https://genome.ucsc.edu/) and aligned.

**AMOT**																				
**Human**	L	V	A	H	A	A	R	Q	E	P	Q	G	Q	E	I	Q	S	E	N	L
**Mouse**	L	V	A	H	-	A	R	Q	E	P	Q	G	Q	E	I	Q	S	E	N	-
**Dog**	L	V	A	H	A	A	R	Q	E	P	Q	G	Q	E	I	Q	-	E	N	-
**Xenopus**	L	V	-	H	A	A	R	Q	E	P	Q	G	Q	E	I	Q	-	E	N	-
**Zebrafish**	-	V	-	H	-	A	R	Q	E	P	Q	G	Q	E	L	Q	-	-	-	-
**AMOTL1**																				
**Human**	M	V	Y	Q	S	A	R	Q	E	P	Q	G	Q	E	H	Q	V	D	N	T
**Mouse**	M	V	Y	Q	S	A	R	Q	E	P	Q	G	Q	E	H	Q	G	D	N	T
**Dog**	M	V	Y	Q	S	A	R	Q	E	P	Q	G	Q	E	H	Q	V	D	N	T
**Xenopus**	M	V	-	Q	S	A	R	Q	E	P	Q	G	Q	E	H	-	-	D	N	T
**Zebrafish**	-	-	-	-	-	-	R	Q	E	P	Q	G	Q	E	H	Q	-	D	-	-
**AMOTL2**																				
**Human**	V	L	Q	Q	A	T	R	Q	E	P	Q	G	Q	E	H	Q	G	G	E	N
**Mouse**	V	L	Q	Q	A	T	R	Q	E	P	Q	G	Q	E	H	Q	G	G	E	T
**Dog**	V	L	Q	Q	A	T	R	Q	E	P	Q	G	Q	E	H	Q	G	G	E	S
**Xenopus**	-	-	-	-	-	-	R	Q	E	P	Q	G	Q	E	-	-	-	-	-	-
**Zebrafish**	-	-	Q	-	-	-	R	Q	E	P	Q	G	Q	E	H	Q	G	-	-	-
